# Cross-residual knowledge graph learning for robust multi-trait gene–trait prioritization in rice

**DOI:** 10.3389/fpls.2026.1883598

**Published:** 2026-08-13

**Authors:** Jingchao Wang, Xiaodan Cui, Xiaoqin Fu, Jiaqi Mao, Yongzhi Zhang, Fangfang Shan, Zhenglong Zhou, Sirui Xing, Xuelin Hu

**Affiliations:** 1 School of Computer Science, Zhongyuan University of Technology, Zhengzhou, China; 2 Liuzhou Railway Vocational Technical College, Liuzhou, Guangxi, China; 3 China Construction Urban Science Municipal Survey and Design Co., Ltd., Lanzhou, Gansu, China

**Keywords:** candidate gene prioritization, crop genetics, gene-trait association, graph neural network, heterogeneous graph learning, knowledge graph, multi-trait prioritization, rice

## Abstract

**Introduction:**

Prioritizing genes associated with agronomic traits remains challenging because relevant evidence is distributed across heterogeneous biological resources and curated gene-trait associations are incomplete.

**Methods:**

We formulate multi-trait crop gene prioritization as gene-trait link prediction on a rice-centered heterogeneous knowledge graph, where each candidate pair represents a graph-supported ranking hypothesis rather than an independent biological validation. We introduce a cross-residual knowledge-graph learning framework that keeps structural and relation-aware propagation in separate branches while allowing layer-wise residual exchange between them. This design lets broad topology and typed biological context refine each other during message passing, and residual filtering or transformation controls weak cross-branch corrections.

**Results:**

Under random and cold-gene evaluation protocols, Cross + JK variants provide consistent information gain within strict dual-branch architectural controls, remain near the top across the broader benchmark, and exhibit small seed-to-seed standard deviations on random-split ranking metrics. The default cold-gene split shows that feature-only MLP is a strong prior-driven baseline, whereas negative-ratio sensitivity shows that MLP becomes unstable when the sampled candidate distribution shifts and Cross + JK variants provide robust information gain in more uncertain candidate-ranking regimes. Broader comparisons with feature-only, heterogeneous graph, graph-Transformer, and KG-embedding baselines show that feature priors and alternative graph models remain strong in some regimes.

**Discussion:**

The results support cross-residual learning as a robust, condition-dependent strategy for integrating multi-source biological evidence in rice gene-trait prioritization.

## 1 Introduction

Crop gene prioritization increasingly depends on combining curated gene–trait associations with pathway, protein interaction, domain, and quantitative trait locus (QTL) evidence. In practice, these resources are distributed across separate databases and often analyzed in isolation, which is especially problematic for multi-trait prioritization because useful signal typically arises from several weak but complementary evidence channels. Although recent crop data platforms have made such integration more feasible (Hassani-Pak et al., 2021; Larmande et al., 2025; Goodstein et al., 2012; Tello-Ruiz et al., 2021; Yonemaru et al., 2010), end-to-end predictive frameworks that learn directly from heterogeneous crop relations remain limited.

Knowledge graphs provide a natural representation because they can jointly encode genes, traits, proteins, pathways, domains, and QTLs together with typed biological relations. Crop-oriented studies and resources such as KnetMiner, AgroLD, and recent crop KG work show the value of organizing heterogeneous agronomic evidence in graph form (Zhang et al., 2024; Hassani-Pak et al., 2021; Larmande et al., 2025). However, many existing efforts emphasize graph construction or evidence retrieval rather than predictive representation learning for gene–trait association modeling.

This gap is also methodological. Graph neural networks (GNNs) provide a flexible way to learn from biological graphs, with GCN, GraphSAGE, and GAT as common general-purpose baselines and R-GCN as a standard relation-aware baseline (Kipf and Welling, 2017; Hamilton et al., 2017; Velickovic et al., 2018; Schlichtkrull et al., 2018). Relation-aware attention and scalable local–global graph Transformers provide stronger tests of whether typed attention or long-range interaction is sufficient for this task (Busbridge et al., 2019; Rampasek et al., 2022). Related work on deeper propagation, link prediction, and knowledge graph completion provides the broader technical foundation (Xu et al., 2019; Rong et al., 2020; Chen et al., 2020; Dwivedi et al., 2023; Zhang and Chen, 2018; Bordes et al., 2013; Nickel et al., 2011; Yang et al., 2015; Trouillon et al., 2016; Dettmers et al., 2018; Sun et al., 2019). Recent inductive knowledge graph completion research has further combined ontology signals, local subgraph reasoning, and prompt-based conditioning to support prediction involving unseen entities (Wang et al., 2026). However, most of these methods are not designed for crop graphs that combine molecular, functional, and breeding-oriented evidence in one prediction space.

For crop gene prioritization, this creates two practical challenges. First, relation groups differ markedly in biological specificity, scale, and noise level. Second, directly applying a single-stream graph model can blur topological and relation-specific signals, particularly under cold-gene evaluation where positive gene–trait supervision is withheld for disjoint genes. These considerations motivate a branch-separated design in which broad structural propagation and relation-aware propagation are modeled separately but still allowed to interact.

To address this problem, we construct a rice-centered heterogeneous knowledge graph benchmark and investigate a cross-residual knowledge-graph learning framework within a broader model comparison. Here, multi-trait prioritization is used in a narrow operational sense: one gene may be ranked for multiple traits, but the model does not explicitly learn trait–trait dependency structure. Each candidate gene–trait pair is therefore a prioritization hypothesis supported or unsupported by the current graph, not a direct claim of experimentally validated causality. Under a unified five-seed protocol, we evaluate feature-only, homogeneous graph, heterogeneous graph, graph-transformer, KG-embedding, residual-variant, and architectural-control models on both a standard random split and a stricter cold-gene split. We interpret the results as evidence about the settings in which cross-residual graph learning helps integrate heterogeneous biological context, while also reporting regimes where feature priors or other graph models are stronger. The main contributions are as follows.

1. We formulate multi-trait crop gene prioritization as heterogeneous gene–trait link prediction on a rice-centered biological knowledge graph and clarify the biological meaning and limits of candidate pairs in this setting.
2. We isolate cross-residual exchange, JK-style aggregation, and residual filtering or transformation in strict controls. Cross + JK variants provide consistent information gains within the dual-branch family, maintain favorable ranking behavior as the negative sampling ratio changes, benefit from GO-based context augmentation, and maintain small random-split standard deviations across the main ranking and threshold metrics.
3. We establish a reproducible benchmark that compares feature-only, graph, graph-transformer, KGembedding, architectural-control, relation-ablation, feature-ablation, and statistical-significance results under random and cold-gene supervision.

## 2 Materials and methods

### 2.1 Task formulation

We formulate the task as heterogeneous link prediction between gene nodes and trait nodes. Here, multi-trait prioritization means that one gene may be ranked for multiple agronomic traits; the model does not explicitly learn a phenotype–phenotype graph. Let G=(V,ℰ,ℛ,ϕ,ψ) denote a heterogeneous graph, where *ϕ* maps nodes to six biological types, T={gene, trait, protein, pathway, domain, qtl}, and *ψ* maps edges to typed biological relations. The input feature matrix is $X\in {\mathbb{R}}^{\left|V\right|\times d_{0}}$ with *d*_0_ = 32. Edges encode gene–trait, gene–gene, gene–protein, protein–protein, gene–pathway, gene–domain, QTL–gene, and QTL–trait relations. For a candidate gene–trait pair (*g*, *t*), the score is interpreted biologically as a ranking hypothesis that gene *g* may be relevant to trait *t* under the evidence encoded in the current graph. It is not an independent validation of the association. The model predicts

(1) $${\hat{y}}_{g,t}=f_{\theta }(g,t|G),$$

In Equation 1, where *f_θ_* is a graph-based scoring model and ${\hat{y}}_{g,t}\in \left[0,1\right]$. In the remainder of this section, *f_θ_* is instantiated by the encoder–decoder architecture described below.

For training, the random split samples positive gene–trait edges uniformly before matched negative sampling, whereas the cold-gene split partitions genes into disjoint training, validation, and test subsets before constructing the corresponding edges. The cold-gene protocol withholds positive gene–trait supervision for validation and test genes, but retains those genes and their non-supervision biological context in the graph. Sampled unobserved pairs are treated as negative supervision surrogates, so evaluation measures discrimination against sampled unknown pairs rather than discrimination against experimentally verified non-associations.

### 2.2 Graph construction and data integration

The experimental graph is centered on rice and integrates curated gene–trait associations, protein interaction context, pathway membership, domain evidence, QTL evidence, and GO annotations after local identifier harmonization. Detailed public source names, database endpoints, and access information are reported in the Data Availability Statement so that the Methods section can focus on graph construction rules, supervision control, modeling, and evaluation.

#### 2.2.1 Identifier harmonization and trait normalization

All resources are mapped to a unified gene namespace before graph assembly. funRiceGenes locus identifiers and aliases are used as the primary reference. STRING aliases are mapped back to genes, KEGG identifiers are resolved to the same namespace, and Q-TARO gene mentions are matched with the same symbol/alias pool. Trait labels from funRiceGenes and Q-TARO are normalized by lower-casing, synonym merging, and blacklist-based removal of overly generic descriptors. This reduces synonym redundancy, but graph semantics and labels still depend strongly on these normalization and grouping rules. Fine-grained phenotype boundaries may therefore differ from alternative trait ontologies or manual curation schemes.

#### 2.2.2 Edge construction rules

The final graph contains eight directed biological relation families before reverse-edge augmentation: gene– trait, gene–gene, gene–protein, protein–protein, gene–pathway, gene–domain, QTL–gene, and QTL–trait. Gene–trait edges provide the supervision signal. Gene–protein and protein–protein edges are obtained from STRING after identifier resolution and confidence filtering. Gene–pathway edges are derived from KEGG membership tables. Gene–domain edges are obtained from domain/family annotations, and gene–gene edges are induced from shared domain evidence under the configured overlap threshold. QTL–trait edges are derived from Q-TARO trait descriptors, and QTL–gene edges are obtained by combining alias matches with interval-based expansion under the configured interval filtering rule. Reverse edges are added to all relation types to support bidirectional message passing.

**Figure 1:**
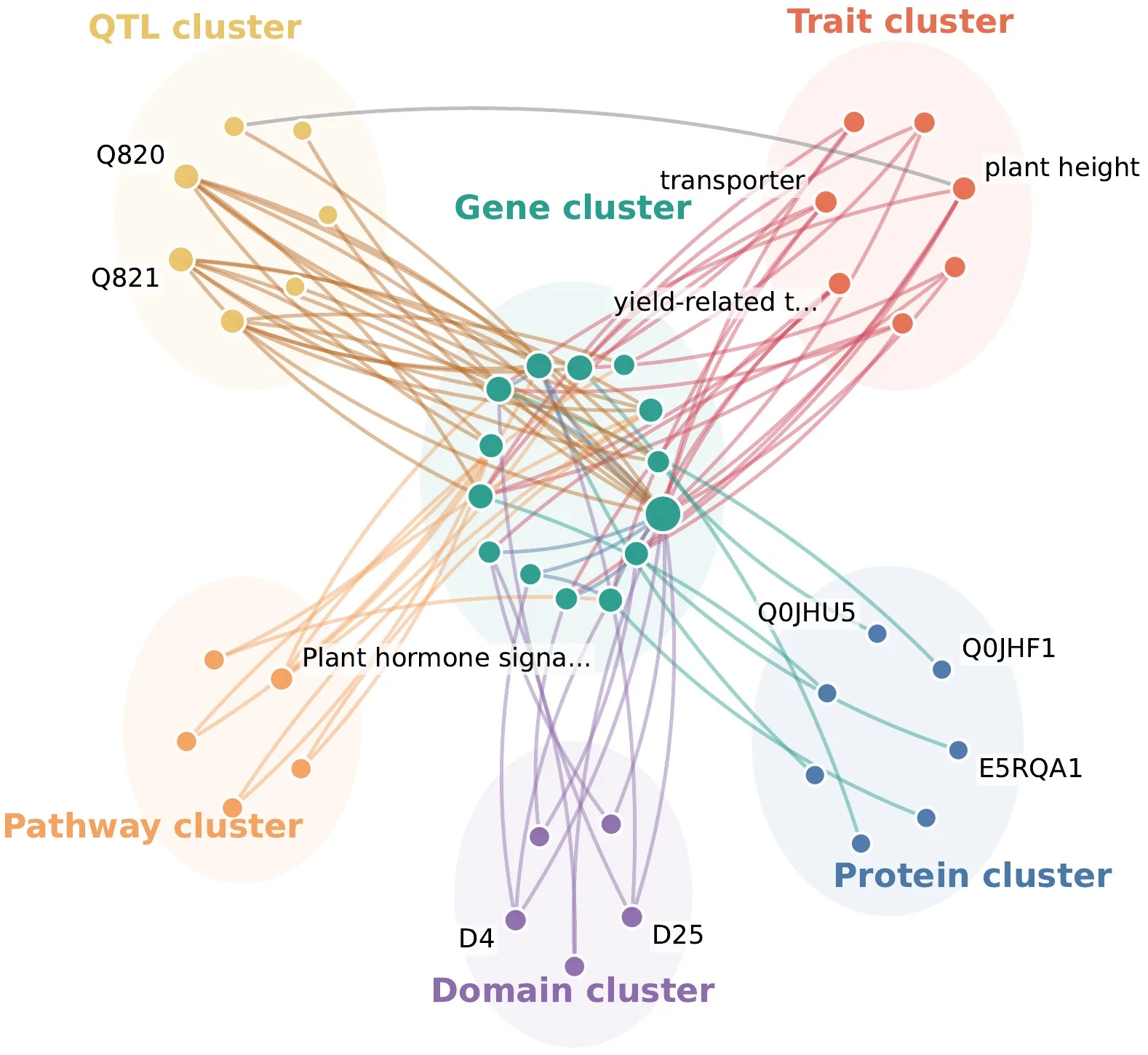
Sampled structural overview of the rice knowledge graph. Gene nodes occupy the center because prediction is defined over gene–trait associations, while trait, protein, pathway, domain, and QTL nodes form typed evidence neighborhoods. Edge colors indicate distinct biological relation families, and node size is proportional to sampled local degree. The panel is schematic rather than an exhaustive rendering of the 10,706-node network.

**Figure 1** summarizes the integrated graph schema using a readability-oriented sampled subgraph. The graph is strongly gene-centered, and different relation groups contribute distinct biological neighborhoods: trait nodes provide supervision, protein nodes capture interaction context, pathway and domain nodes encode functional membership, and QTL nodes inject positional evidence. The figure is illustrative rather than exhaustive.

#### 2.2.3 Leakage control and supervision split

Gene–trait edges define the primary supervision targets. Held-out positive target edges are excluded from the training supervision, while non-supervision biological context for the same nodes is retained for message passing. This protocol is intended to reduce direct supervision leakage while preserving the heterogeneous evidence needed for prioritization; as with any integrated biological knowledge graph, related public resources may still provide indirect contextual signals, and the results are interpreted with this boundary in mind.

After filtering and integration, the graph used in the finalized experiments contains 10,706 nodes with 6 node types and 16 directed relation types after reverse-edge augmentation. The node composition is 4,421 genes, 1,025 traits, 3,774 proteins, 150 pathways, 285 domains, and 1,051 QTL nodes. The raw integrated tables contain 15,763 gene–trait edges, 3,750 gene–gene edges, 3,782 gene–protein edges, 21,826 protein–protein edges, 2,933 gene–pathway edges, 1,095 gene–domain edges, 19,196 QTL–gene edges, 1,030 QTL–trait edges, and 22,974 GO annotations retained for auxiliary evidence tracing.

**Figure 2:**
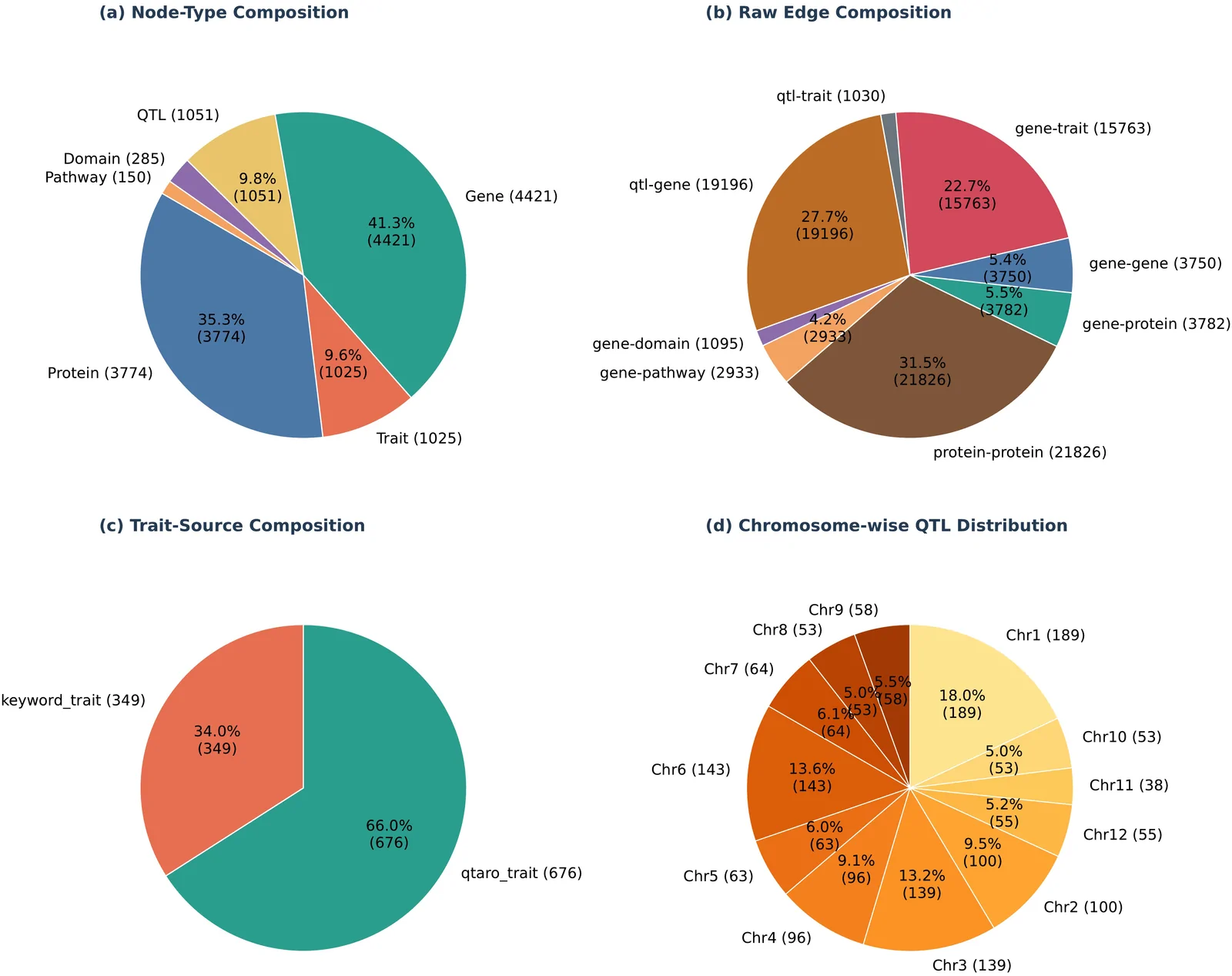
Global statistics of the rice knowledge graph. **(a)** reports node-type composition, **(b)** reports raw edge composition before reverse-edge augmentation, **(c)** reports normalized trait-source composition, and **(d)** reports chromosome-wise QTL distribution. Together, they highlight the scale imbalance and heterogeneous coverage of the graph components.

The global composition of this integrated graph is summarized in **Figure 2**. Panel (a) shows that genes and proteins account for most graph entities. Panel (b) shows that protein–protein and QTL–gene edges are the largest raw relation groups. Panel (c) shows that trait nodes combine Q-TARO and keyword-derived sources. Panel (d) shows that QTL evidence is distributed across all rice chromosomes. Together, these statistics highlight that relation groups differ in both meaning and scale.

**Figure 3:**
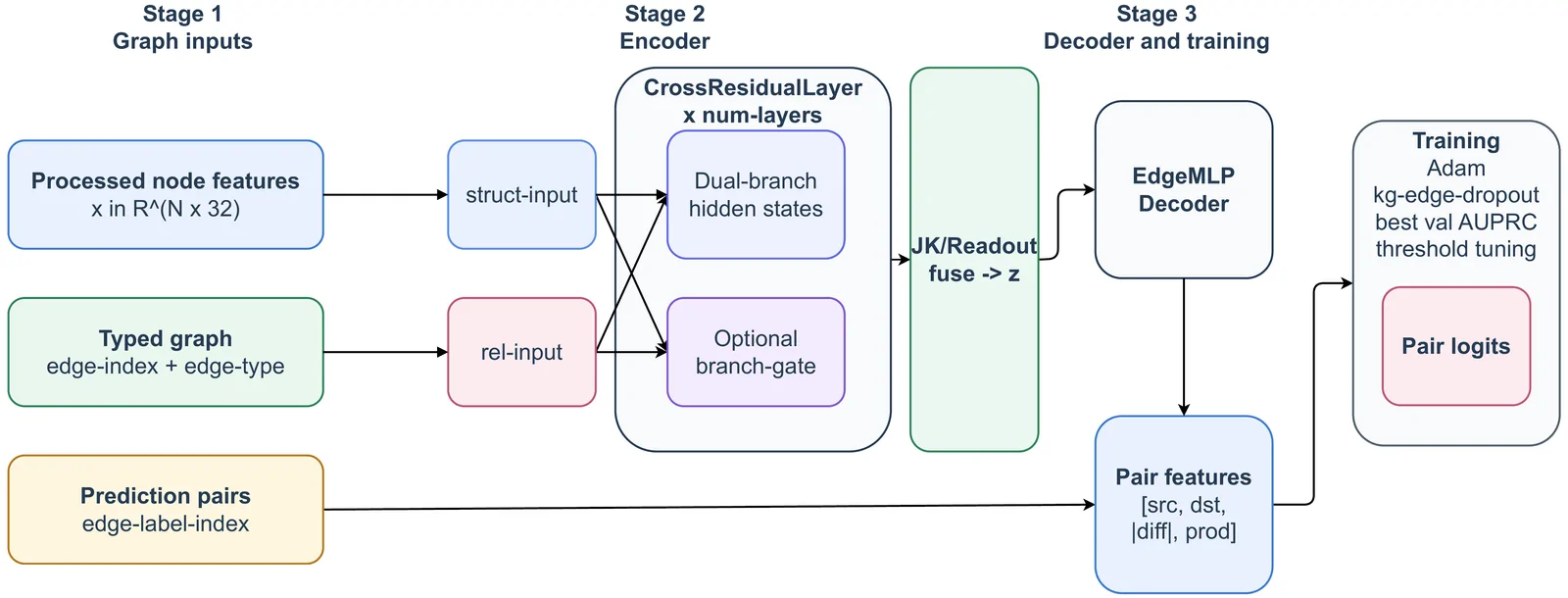
Overview of the prediction pipeline. Stage 1 prepares processed node features, typed graph structure, and prediction pairs. Stage 2 applies the dual-branch encoder with structural and relation-aware propagation plus layer-wise cross-residual exchange before a standard final readout. Stage 3 computes pairwise features, decodes gene–trait scores, and optimizes the model under the finalized training protocol.

With the graph schema, relation composition, and supervision split defined, the full modeling workflow can now be described from inputs to training. **Figure 3** serves as the method-level overview that connects the remaining subsections. In Stage 1, processed node features, typed graph structure, and prediction pairs are prepared as model inputs. In Stage 2, the encoder maps node features into structural and relation-aware branches, applies the layer-wise cross-residual interaction, and then uses a standard readout fusion step to form the final node embedding. In Stage 3, an edge decoder computes pairwise logits and the model is optimized under the finalized training protocol. The following subsections unpack these three stages in the same order.

### 2.3 Node features

The first part of Stage 1 in **Figure 3** is the construction of processed node features. Each node is represented in a shared 32-dimensional feature space. The finalized feature vector combines node-type identity, logarithmic degree, isolation flags, and hashed membership signals derived from domain, pathway, QTL, and protein-mapping evidence. Although GO annotations are retained in the integrated evidence tables, the fixed 32-dimensional configuration used in the finalized experiments allocates no remaining hashed feature width to GO. This detail is important for interpreting the feature-ablation results: the strongest feature-only baselines are driven mainly by type, degree, and non-GO membership priors.

### 2.4 Knowledge graph-enhanced cross-residual encoder

Stage 2 of **Figure 3** is the dual-branch encoder. The evaluated encoder contains two message-passing branches. The first branch is a structural branch based on GCN propagation (Kipf and Welling, 2017) to capture broad topological context. The second branch is a relation-aware branch based on R-GCN propagation (Schlichtkrull et al., 2018) to preserve heterogeneous biological semantics. Let $H_{s}^{\left(\ell \right)},H_{r}^{\left(\ell \right)}\in {\mathbb{R}}^{\left|V\right|\times d}$ denote thestructural and relation-aware hidden states at layer ℓ, where *d* is the hidden dimension.

The separate learnable input projections used to initialize the two branches before graph propagation are defined in Equation 2 as follows:

(2) $$H_{s}^{\left(0\right)}={\text{Proj}}_{s}\left(X\right),\;H_{r}^{\left(0\right)}={\text{Proj}}_{r}\left(X\right),$$

The structural branch computes with The structural branch message is defined in Equation 3 as.

(3) $$M_{s}^{\left(\ell \right)}={\text{GCN}}^{\left(\ell \right)}\left(H_{s}^{\left(\ell \right)},ℰ\right),$$

and the relation-aware branch computes with and the relation-aware branch message is defined in Equation 4 as.

(4) $$M_{r}^{\left(\ell \right)}={\text{RGCN}}^{\left(\ell \right)}\left(H_{r}^{\left(\ell \right)},ℰ,ℛ\right).$$

To avoid prematurely collapsing the two signals, we keep branch-internal residual accumulation and introduce cross-residual exchange. The structural-branch update is given in Equation 5, and the relation-aware branch update is given in Equation 6:

(5) $${\tilde{H}}_{s}^{\left(\ell +1\right)}=H_{s}^{\left(\ell \right)}+M_{s}^{\left(\ell \right)}+\phi_{r\to s}^{\left(\ell \right)}\left(M_{r}^{\left(\ell \right)}\right),$$

(6) $${\tilde{H}}_{r}^{\left(\ell +1\right)}=H_{r}^{\left(\ell \right)}+M_{r}^{\left(\ell \right)}+\phi_{s\to r}^{\left(\ell \right)}\left(M_{s}^{\left(\ell \right)}\right),$$

where $\phi_{r\to s}^{\left(\ell \right)}$ and $\phi_{s\to r}^{\left(\ell \right)}$ are learnable linear transformations that project messages across branches. The updated states are then normalized, activated, and regularized with dropout; the normalized structural state is given in Equation 7, and the normalized relation-aware state is given in Equation 8:

(7) $$H_{s}^{\left(\ell +1\right)}=\text{Dropout}\left(\sigma \left(\text{LN}\left({\tilde{H}}_{s}^{\left(\ell +1\right)}\right)\right)\right),$$

(8) $$H_{r}^{\left(\ell +1\right)}=\text{Dropout}\left(\sigma \left(\text{LN}\left({\tilde{H}}_{r}^{\left(\ell +1\right)}\right)\right)\right),$$

where LN is layer normalization and *σ* is a nonlinear activation.

After the layer-wise encoder updates, a final readout is required to convert the two branch states into a single embedding for edge scoring. Denoting this readout combiner by *γ*(·,·), the final node representation is obtained by combining the last-layer branch outputs and projecting them into a shared embedding space and the updated states are then normalized, activated, and regularized with dropout; the normalized structural state is given in Equation 7, and the normalized relation-aware state is given in Equation 8:

(9) $$Z=\psi_{\text{fuse}}\left(\gamma \left(H_{s}^{\left(L\right)},H_{r}^{\left(L\right)}\right)\right),$$

In Equation 9, where *ψ*_fuse_ is a learned linear projection and *L* is the number of graph layers. In practice, γ(A,B)=[A∥B] when branch gating is disabled and corresponds to gated concatenation when branch gating is enabled. This output fusion step is not the cross-residual interaction mechanism; it is a standard readout required by any dual-branch encoder. The evaluated cross-residual component is the intermediate exchange in the layer updates above.

#### 2.4.1 Relation to existing skip and fusion mechanisms

The cross-residual design is intended as a task-oriented extension of established skip-connection and fusion ideas for this crop knowledge-graph setting. Unlike ordinary residual connections, each branch receives both its own residual state and a transformed message from the other branch. Unlike Jumping Knowledge (Xu et al., 2018) or pure late fusion, branch interaction occurs during propagation rather than only at the final readout. We therefore emphasize its empirical value for the present crop-oriented prioritization task while also comparing it with closely related aggregation and fusion controls.

### 2.5 Architectural control variants

To isolate the effect of cross-branch interaction, we additionally evaluate parameter-comparable controls built on the same dual-branch backbone. In late fusion, the two branches are propagated independently and concatenated only after the final layer. In gated-fusion-only, the final branch embeddings are combined through a learned fusion gate without intermediate cross-residual correction. In JK-style, following the multi-layer aggregation principle of Jumping Knowledge Networks (Xu et al., 2018), branch outputs from all layers are concatenated before the final projection. We also evaluate a strict Cross × JK factorial design with the final branch gate disabled: cross-residual only, JK only, Cross + JK, and filtered Cross + JK. This separates intermediate cross-branch exchange from multi-layer readout aggregation.

### 2.6 Residual transforms

The residual comparison keeps the encoder topology fixed and varies the residual transform *T*(·) applied before residual accumulation. Let **r** denote either a branch-internal residual state or a cross-branch exchanged message. This filtering stage was introduced because plain residual exchange proved empirically sensitive to weak correction signals. The residual-enhanced structural update is given in Equation 10, and the residual-enhanced relation-aware update is given in Equation 11.

(10) $${\tilde{H}}_{s}^{\left(\ell +1\right)}=M_{s}^{\left(\ell \right)}+T_{s}\left(H_{s}^{\left(\ell \right)}\right)+T_{r\to s}\left(\phi_{r\to s}^{\left(\ell \right)}\left(M_{r}^{\left(\ell \right)}\right)\right),$$

(11) $${\tilde{H}}_{r}^{\left(\ell +1\right)}=M_{r}^{\left(\ell \right)}+T_{r}\left(H_{r}^{\left(\ell \right)}\right)+T_{s\to r}\left(\phi_{s\to r}^{\left(\ell \right)}\left(M_{s}^{\left(\ell \right)}\right)\right).$$

**Figure 4:**
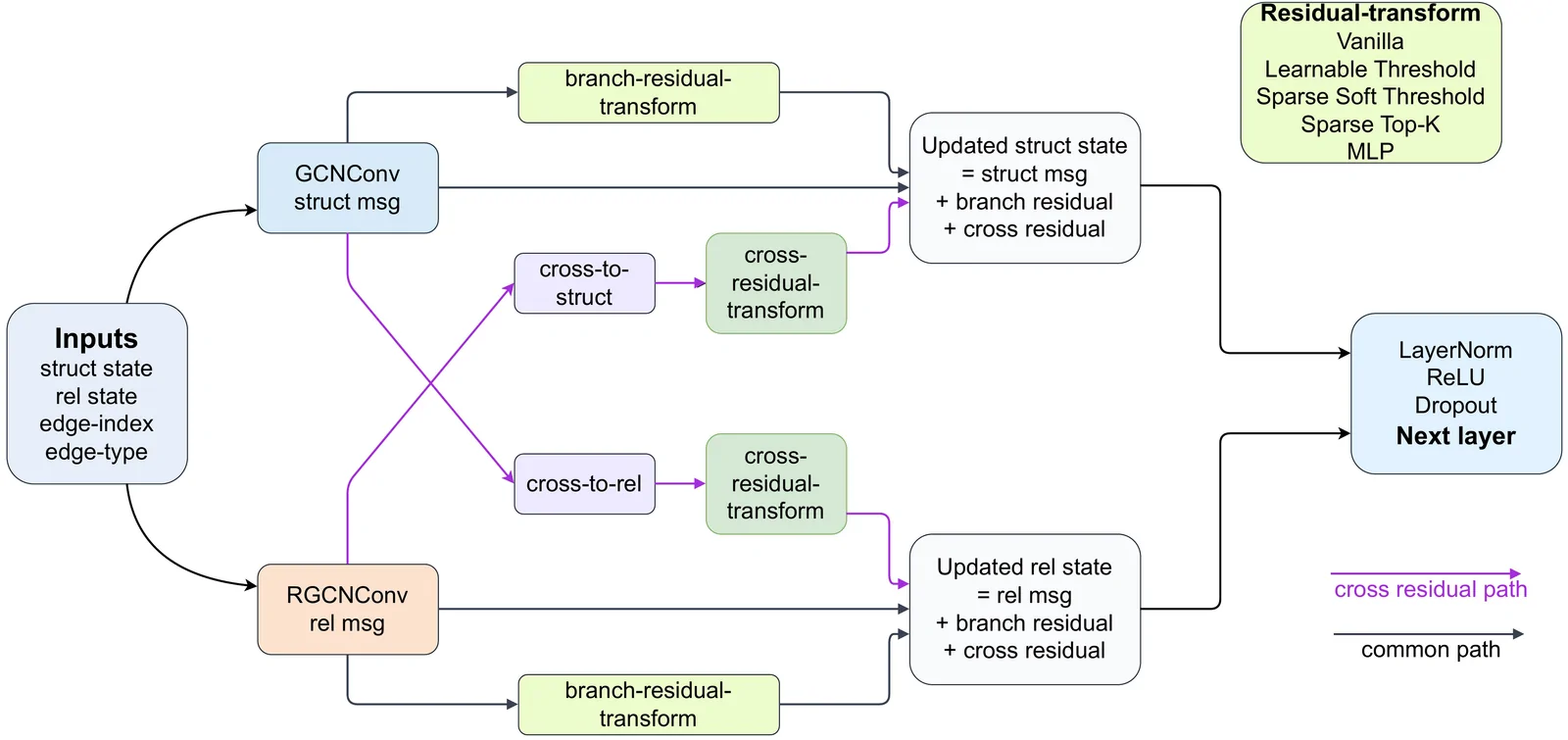
Layer-level view of the residual mechanisms evaluated in this study. Within one dual-branch encoder layer, the structural branch applies GCN-based propagation and the relation-aware branch applies R-GCN-based propagation, after which branch residuals and cross-branch exchanged messages are transformed before accumulation. The residual comparison evaluates vanilla, learnable-threshold, sparse soft-threshold, sparse Top-K, and MLP residual transforms within this layer.

The vanilla residual transform is defined in Equation 12, the learnable-threshold residual transform in Equation 13, the soft-threshold residual transform in Equation 14, the Top-K residual transform in Equation 15, and the MLP residual transform in Equation 16.

(12) $$T_{\text{vanilla}}\left(r\right)=r,$$

(13) $$T_{\text{learn}}\left(r\right)=\text{sign}\left(r\right)⊙\max\left(\left|r\right|-\tau ,0\right),$$

(14) $$T_{\text{soft}}\left(r\right)=\text{sign}\left(r\right)⊙\max\left(\left|r\right|-\lambda ,0\right),$$

(15) $$T_{\text{topk}}\left(r\right)=m_{k}\left(r\right)⊙r,$$

(16) $$T_{\text{mlp}}\left(r\right)={\text{MLP}}_{\text{res}}\left(r\right),$$

where *τ* is a learnable non-negative threshold vector implemented through a softplus parameterization, *λ* is a fixed soft-threshold constant, $m_{k}\left(r\right)$ retains only the Top-K residual magnitudes per node, and MLP_res_ is a small learnable residual transform. The thresholded, sparse, and MLP variants aim to suppress or reshape weak residual noise while preserving informative cross-branch corrections. **Figure 4** illustrates the residual pipeline. When all residual transforms are set to the identity map, the update reduces to the vanilla cross-residual formulation introduced earlier; the residual experiments therefore isolate the effect of filtering rather than changing the surrounding encoder backbone. **Figure 4** provides the layer-level view of this mechanism. The input block corresponds to the branch states and graph tensors entering one encoder layer. The structural path applies GCN-based propagation over edge-index, whereas the relation-aware path applies R-GCN over both edge-index and edge-type. Branch-internal residuals and cross-branch exchanged messages are each passed through a residual transform before accumulation, after which the updated states are normalized, activated, regularized, and forwarded to the next layer. In this sense, **Figure 3** explains where the encoder sits in the full pipeline, while **Figure 4** explains how one encoder layer operates internally.

### 2.7 Prediction head and learning objective

Stage 3 of **Figure 3** begins with the prediction head. For a gene–trait pair (*g,t*), the final score is computed by an edge decoder applied to the corresponding node embeddings:

(17) $${\hat{y}}_{g,t}=\sigma \;\left(\text{MLP}\left(\left[z_{g}∥z_{t}∥|z_{g}-z_{t}|∥z_{g}⊙z_{t}\right]\right)\right),$$

In Equation 17, where **z***_g_* and **z***_t_* are the final gene and trait embeddings, | · | denotes element-wise absolute difference, and ⊙ denotes element-wise multiplication. Training uses mean binary cross-entropy over observed positive gene–trait associations and randomly sampled negative pairs:

(18) $${ℒ}_{\text{GT}}=-\frac{1}{\left|P_{tr}\right|+\left|N_{tr}\right|}\left[\sum_{\left(g,t\right)\in P_{\text{tr}}}\text{log }{\hat{y}}_{g,t}+\sum_{\left(g,t\right)\in N_{\text{tr}}}\text{log }(1-{\hat{y}}_{g,t})\right],$$

In Equation 18, where $P_{tr}$ and $N_{tr}$ are the positive and negative training sets. Sampled negatives are supervision surrogates rather than verified biological non-associations.

#### 2.7.1 Relation-balanced auxiliary multi-task objective

For the auxiliary multi-task experiments, non-supervision relation families are optimized as held-out typed edge-completion tasks. Let ${ℛ}_{\text{aux}}$ denote the auxiliary relation set excluding gene–trait supervision, including gene–gene, gene–protein, protein–protein, gene–pathway, gene–domain, QTL–gene, and QTL– trait completion. Forward and reverse edge types are treated as separate directed prediction tasks, yielding 14 directed auxiliary losses. Within each relation, known edges are split with seed 2026 into messagepassing context, auxiliary training targets, validation targets, and test targets. Approximately 60% of edges remain as context, 20% are auxiliary training positives, and 10% each are reserved for validation and test. Auxiliary target edges and their paired reverse edges are removed from the message-passing graph. Negative pairs are sampled within the correct source and destination node types and filtered against all known positive edges of that relation. A relation-specific bilinear decoder gives scores ${\hat{y}}_{u,v}^{\left(r\right)}$, and the normalized relation loss is

(19) $${ℒ}_{r}=-\frac{1}{\left|P_{r}\right|+\left|N_{r}\right|}\left[\sum_{\left(u,v\right)\in P_{r}}\text{log }{\hat{y}}_{u,v}^{\left(r\right)}+\sum_{\left(u,v\right)\in N_{r}}\text{log }\left(1-{\hat{y}}_{u,v}^{\left(r\right)}\right)\right].$$

Equation 19 defines the normalized auxiliary loss for relation *r*. The relation-balanced true multi-task objective is then

(20) $${ℒ}_{\text{total}}={ℒ}_{\text{GT}}+\lambda_{\text{aux}}\frac{1}{\left|{ℛ}_{aux}\right|}\sum_{r\in {ℛ}_{\text{aux}}}{ℒ}_{r},$$

In Equation 20, where ${ℒ}_{\text{GT}}$ is the gene–trait loss above and λ_aux_ controls the auxiliary contribution. This formulation keeps gene–trait prioritization as the primary task while preventing frequent auxiliary relations from dominating the multi-task signal. Validation and test gene–trait positives are never used as auxiliary positives, and held-out auxiliary targets are not visible during message passing.

### 2.8 Transformer baseline configurations

To strengthen the heterogeneous graph comparison, we include heterogeneous attention and graphTransformer variants under the same feature, decoder, optimization, and split protocol. HAN (Wang et al., 2019) and HGT (Hu et al., 2020) provide established heterogeneous graph baselines. RGAT (Busbridge et al., 2019) applies relation-conditioned graph attention to typed edges, with block-diagonal relation transforms to keep edge-wise attention tractable on the full rice graph. The GraphGPS-style variant (Rampasek et al., 2022) combines a local R-GCN operator with global Performer self-attention, providing linear-complexity global interaction rather than dense quadratic attention over all 10,706 nodes. These neural baselines use two 128-dimensional layers and the same edge MLP decoder unless otherwise stated in **Table 1**.

**Table 1:** Main training and reproducibility settings used throughout the finalized experiments unless otherwise specified.

Item	Setting
Hardware	Single NVIDIA RTX 3090 GPU
Hidden dimension/output dimension	128/128
Number of graph layers	2
Dropout	0.2
Optimizer	Adam
Learning rate	1 × 10^−3^
Weight decay	1 × 10^−4^
Knowledge-edge dropout	0.15
Early stopping	patience 5, selected by validation AUPRC
Threshold tuning	validation macro-F1 over a fixed threshold grid
Negative sampling ratio (default)	1:1
Primary main-table seeds	42, 52, 62, 72, 82

### 2.9 Training protocol

The final part of Stage 3 in **Figure 3** is the training protocol. All reported results follow a unified finalized protocol. Model selection is based on validation AUPRC, the best checkpoint is saved, the decision threshold *τ*^∗^ is tuned on the validation set by maximizing macro-F1, and training-time knowledge-edge dropout is applied to non-supervision relations. The residual comparison keeps this protocol fixed and varies only the residual transform.

Unless otherwise stated, the main results use the configuration summarized in **Table 1**. This table fixes the capacity, optimization, checkpoint-selection, threshold-selection, negative-sampling, and seed settings used by the main comparisons. The same protocol is shared by the baseline comparison, residual comparison, architectural controls, relation-level ablation, and trait-subset analysis, so differences reported in the results primarily reflect model structure or graph evidence rather than changes in training procedure.

## 3 Results

### 3.1 Evaluation design

We evaluate gene–trait ranking under two complementary protocols. The random split tests edge-level completion when genes have related supervision during training. The cold-gene split withholds positive gene–trait supervision for validation and test genes and is closer to candidate prioritization for less characterized genes. AUPRC is the primary ranking metric because candidate pairs are evaluated against sampled unobserved pairs; AUROC, macro-F1, and accuracy are reported to clarify threshold-independent and thresholded behavior. Unless stated otherwise, values are mean ± standard deviation over five seeds (42, 52, 62, 72, and 82), and paired comparisons use seed-matched tests with Holm correction (Holm, 1979). The negative-ratio experiments use three seeds because each split is rebuilt at five class-imbalance levels.

### 3.2 Main benchmark comparison

**Tables 2**, **3** place the proposed cross-residual models alongside feature-only, homogeneous graph, heterogeneous graph, graph-Transformer, and knowledge-graph embedding baselines. The comparison uses the same processed feature space and the same gene–trait decoder for neural graph models. HAN and HGT are included as heterogeneous graph controls, RGAT provides relation-aware attention, and the GraphGPS-style variant combines local relational propagation with global attention. DistMult, ComplEx, and RotatE are evaluated as typed-edge embedding baselines.

**Table 2:** Core benchmark ranking metrics under random and cold-gene splits.

Model	Random AUPRC	Random AUROC	Cold-gene AUPRC	Cold-gene AUROC
MLP	0.9054 ± 0.0035	0.9291 ± 0.0021	**0.9176 ± 0.0025**	**0.9310 ± 0.0019**
GCN	0.9086 ± 0.0014	0.9335 ± 0.0012	0.8456 ± 0.0133	0.8909 ± 0.0096
GraphSAGE	0.9262 ± 0.0017	0.9427 ± 0.0011	0.8504 ± 0.0125	0.8893 ± 0.0062
GAT	0.9027 ± 0.0028	0.9304 ± 0.0015	0.8395 ± 0.0061	0.8845 ± 0.0032
R-GCN	0.9106 ± 0.0056	0.9359 ± 0.0018	0.8443 ± 0.0092	0.8900 ± 0.0087
HAN	0.8996 ± 0.0134	0.9283 ± 0.0070	0.7762 ± 0.0071	0.8434 ± 0.0034
HGT	0.8848 ± 0.0144	0.9175 ± 0.0069	0.8348 ± 0.0296	0.8826 ± 0.0199
RGAT	**0.9303** ± **0.0033**	**0.9469 ± 0.0014**	0.8557 ± 0.0124	0.8980 ± 0.0069
GraphGPS-style	0.9263 ± 0.0019	0.9457 ± 0.0007	0.9013 ± 0.0129	0.9232 ± 0.0092
DistMult	0.4994 ± 0.0098	0.5005 ± 0.0164	0.5044 ± 0.0063	0.5044 ± 0.0071
ComplEx	0.5022 ± 0.0063	0.5007 ± 0.0072	0.4989 ± 0.0034	0.4986 ± 0.0050
RotatE	0.9297 ± 0.0017	0.9440 ± 0.0009	0.8771 ± 0.0050	0.9170 ± 0.0032
Late fusion	0.9290 ± 0.0015	**0.9469 ± 0.0010**	0.9001 ± 0.0052	0.9283 ± 0.0025
JK only	0.9263 ± 0.0113	0.9442 ± 0.0081	**0.9019 ± 0.0030**	**0.9285 ± 0.0017**
Cross + JK, learnable threshold	**0.9312 ± 0.0010**	**0.9487 ± 0.0006**	**0.9023 ± 0.0029**	0.9282 ± 0.0021
Cross + JK, MLP residual	**0.9301** ± **0.0011**	**0.9479** ± **0.0009**	**0.9050** ± **0.0031**	**0.9294** ± **0.0014**

Values are mean ± standard deviation over five seeds. Bold marks the top three displayed values in each metric column, ties are retained, and underlining marks the column best.

**Table 3:** Core benchmark threshold-dependent metrics under random and cold-gene splits.

Model	Random macro-F1	Random accuracy	Cold-gene macro-F1	Cold-gene accuracy
MLP	0.8635 ± 0.0082	0.8643 ± 0.0076	**0.8591 ± 0.0025**	**0.8596 ± 0.0024**
GCN	0.8697 ± 0.0031	0.8701 ± 0.0033	0.8306 ± 0.0077	0.8325 ± 0.0085
GraphSAGE	0.8749 ± 0.0016	0.8754 ± 0.0015	0.8166 ± 0.0350	0.8181 ± 0.0357
GAT	0.8653 ± 0.0026	0.8656 ± 0.0026	0.8290 ± 0.0110	0.8312 ± 0.0108
R-GCN	0.8719 ± 0.0030	0.8723 ± 0.0029	0.6657 ± 0.1599	0.7010 ± 0.1233
HAN	0.8657 ± 0.0022	0.8666 ± 0.0018	0.8251 ± 0.0106	0.8293 ± 0.0114
HGT	0.8500 ± 0.0072	0.8513 ± 0.0063	0.8299 ± 0.0014	0.8340 ± 0.0010
RGAT	0.8792 ± 0.0011	0.8794 ± 0.0010	0.8009 ± 0.0616	0.8050 ± 0.0565
GraphGPS-style	0.8814 ± 0.0027	0.8817 ± 0.0026	0.7699 ± 0.1369	0.7847 ± 0.1105
DistMult	0.4353 ± 0.0400	0.4979 ± 0.0114	0.4426 ± 0.0703	0.5034 ± 0.0032
ComplEx	0.4332 ± 0.0607	0.4981 ± 0.0076	0.4155 ± 0.0783	0.4988 ± 0.0039
RotatE	0.8728 ± 0.0051	0.8730 ± 0.0049	0.8574 ± 0.0030	0.8579 ± 0.0031
Late fusion	**0.8831 ± 0.0016**	**0.8833 ± 0.0015**	**0.8644 ± 0.0018**	**0.8648 ± 0.0018**
JK only	0.8798 ± 0.0095	0.8801 ± 0.0091	**0.8641 ± 0.0014**	**0.8645 ± 0.0015**
Cross + JK, learnable threshold	**0.8832 ± 0.0015**	**0.8834 ± 0.0015**	0.8478 ± 0.0258	0.8483 ± 0.0250
Cross + JK, MLP residual	**0.8835 ± 0.0020**	**0.8836 ± 0.0020**	0.8548 ± 0.0146	0.8551 ± 0.0146

Values are mean ± standard deviation over five seeds. Bold values indicate the top three displayed values in each metric column; underlined values indicate the best value in each metric column.

For the random-split part of **Table 2**, the strongest ranking results come from models that combine typed graph evidence with expressive readout or embedding mechanisms. RotatE reaches a random AUPRC of 0.9297 ± 0.0017, while Cross + JK with learnable-threshold filtering gives the highest random AUPRC and AUROC in this table (0.9312 ± 0.0010 and 0.9487 ± 0.0006). RGAT, RotatE, late fusion, and the MLP-residual Cross + JK variant are close competitors, showing that the random split is sensitive to several forms of relation-aware evidence integration. Importantly, the proposed Cross + JK variants remain near the top across both ranking and threshold-dependent metrics rather than improving only a single score. Their random-split standard deviations are also consistently small, for example 0.0010 for learnable-threshold AUPRC and 0.0006 for learnable-threshold AUROC, indicating stable learning behavior across repeated seeds. **Table 3** reports the corresponding threshold-dependent metrics; Cross + JK variants are also among the strongest models on random macro-F1 and accuracy, with similarly small seed-to-seed variation.

**Figure 5:**
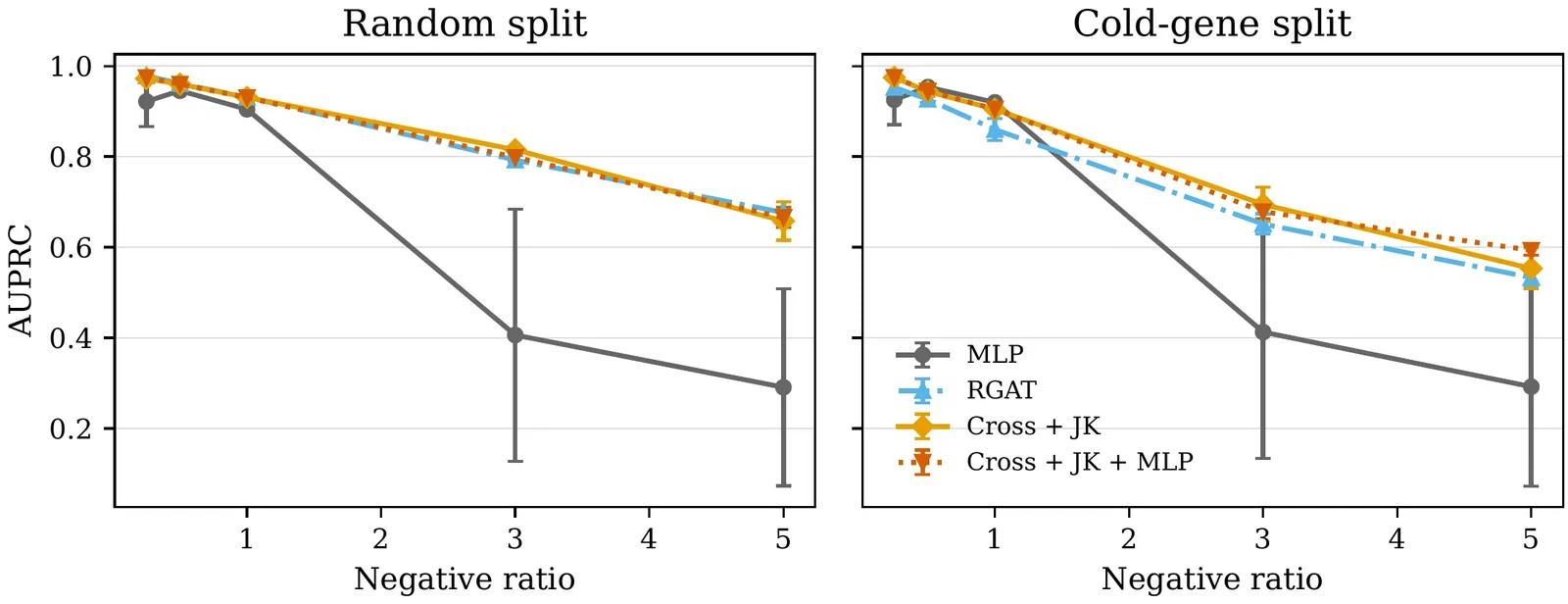
AUPRC sensitivity to the sampled negative ratio. Error bars report standard deviation over three seeds. Cross + JK + MLP denotes the Cross + JK variant with an MLP residual transform. Because AUPRC changes with class prevalence, the main comparison is within each negative-ratio column.

For the cold-gene part of **Tables 2**, **3**, the ranking changes because positive gene–trait supervision for test genes is withheld. The feature-only MLP remains strongest by AUPRC and AUROC under the default evaluation protocol (0.9176 ± 0.0025 and 0.9310 ± 0.0019), indicating that type, degree, and membership-derived features provide strong priors for unseen genes. This result should therefore be interpreted as a strong feature-prior baseline under the default sampled-candidate setting, while the same ordering need not hold across candidate-generation regimes. The negative-ratio sensitivity analysis in **Table 4** and **Figure 5** shows that MLP becomes substantially less stable when the sampled candidate distribution is shifted. In contrast, Cross + JK variants provide robust information gain in more uncertain candidate-ranking regimes, including selected low-ratio cold-gene settings. Within graph-based models, Cross + JK with an MLP residual transform gives the highest cold-gene graph-model AUPRC in the default benchmark (0.9050 ± 0.0031), followed by Cross + JK with learnable-threshold filtering and GraphGPS-style propagation.

**Table 4:** Cold-gene negative-ratio sensitivity.

Negative ratio and model	AUPRC	AUROC
0.25, MLP	0.9254 ± 0.0552	0.7964 ± 0.1464
0.25, RGAT	0.9528 ± 0.0158	0.8922 ± 0.0253
0.25, Cross + JK, learnable threshold	0.9749 ± 0.0015	0.9321 ± 0.0025
0.25, Cross + JK, MLP residual	0.9747 ± 0.0015	0.9320 ± 0.0034
0.50, MLP	0.9535 ± 0.0033	0.9284 ± 0.0027
0.50, RGAT	0.9265 ± 0.0068	0.9031 ± 0.0088
0.50, Cross + JK, learnable threshold	0.9437 ± 0.0035	0.9256 ± 0.0032
0.50, Cross + JK, MLP residual	0.9447 ± 0.0035	0.9269 ± 0.0029
1.00, MLP	0.9197 ± 0.0026	0.9323 ± 0.0007
1.00, RGAT	0.8598 ± 0.0242	0.8970 ± 0.0174
1.00, Cross + JK, learnable threshold	0.9047 ± 0.0027	0.9297 ± 0.0023
1.00, Cross + JK, MLP residual	0.9050 ± 0.0009	0.9297 ± 0.0003
3.00, MLP	0.4126 ± 0.2790	0.6510 ± 0.2303
3.00, RGAT	0.6513 ± 0.0222	0.8914 ± 0.0107
3.00, Cross + JK, learnable threshold	0.6945 ± 0.0385	0.9041 ± 0.0173
3.00, Cross + JK, MLP residual	0.6784 ± 0.0154	0.9038 ± 0.0047
5.00, MLP	0.2920 ± 0.2198	0.6185 ± 0.2056
5.00, RGAT	0.5330 ± 0.0206	0.8908 ± 0.0112
5.00, Cross + JK, learnable threshold	0.5530 ± 0.0450	0.8901 ± 0.0108
5.00, Cross + JK, MLP residual	0.5931 ± 0.0111	0.9099 ± 0.0014

Values are mean ± standard deviation over three seeds.

### 3.3 Cross-residual exchange with JK aggregation

**Figure 6:**
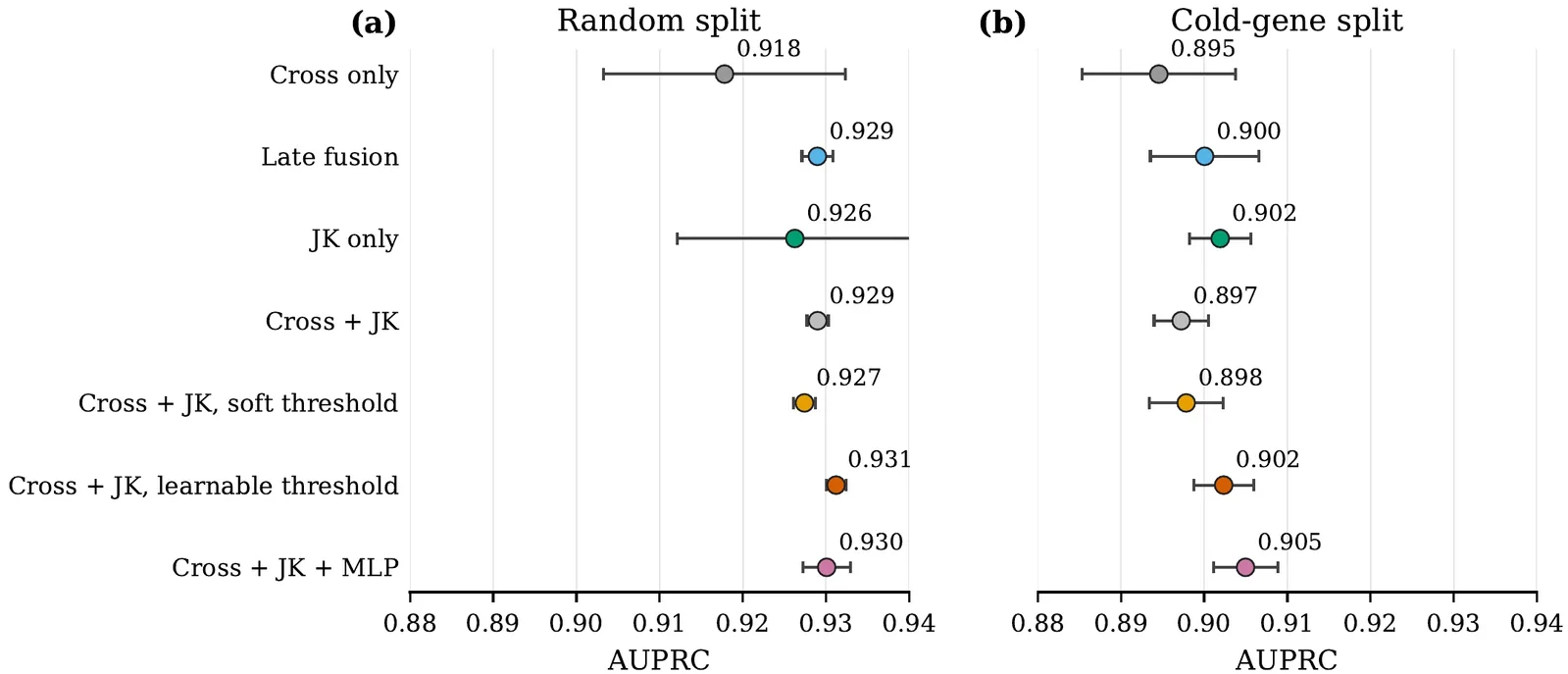
Strict Cross + JK architectural controls. **(a)** Random split and **(b)** cold-gene split. Points and horizontal error bars report mean AUPRC and 95% confidence intervals over five matched seeds.

The main architectural question is whether layer-wise cross-residual exchange adds value beyond JKstyle multi-layer aggregation and residual filtering. **Table 5** reports a strict factorial control in which the two-branch GCN/R-GCN backbone, decoder, optimizer, and final branch gate are fixed. Cross only, JK only, Cross + JK, and filtered or transformed Cross + JK differ only in intermediate exchange, multi-layer aggregation, and residual filtering or transformation. **Figure 6** visualizes the corresponding AUPRC values and 95% confidence intervals for these strict architectural controls under the random and cold-gene splits.

**Table 5:** Ranking metrics for strict Cross × JK architectural controls over five seeds.

Architecture	Random AUPRC	Random AUROC	Cold AUPRC	Cold AUROC
Cross only, vanilla	0.9178 ± 0.0117	0.9407 ± 0.0059	0.8945 ± 0.0074	0.9245 ± 0.0036
JK only	0.9263 ± 0.0113	0.9442 ± 0.0081	**0.9019** ± **0.0030**	**0.9285** ± **0.0017**
Cross + JK, vanilla	**0.9290** ± **0.0010**	0.9468 ± 0.0007	0.8972 ± 0.0026	0.9261 ± 0.0012
Cross + JK, sparse soft threshold	0.9274 ± 0.0011	0.9462 ± 0.0005	0.8978 ± 0.0036	0.9266 ± 0.0012
Cross + JK, learnable threshold	**0.9312 ± 0.0010**	**0.9487 ± 0.0006**	**0.9023** ± **0.0029**	0.9282 ± 0.0021
Cross + JK, MLP residual	**0.9301** ± **0.0023**	**0.9472** ± **0.0013**	**0.9050 ± 0.0031**	**0.9294 ± 0.0014**

Bold values indicate the top three displayed values in each metric column; underlined values indicate the best value in each metric column.

In the random-split half of **Table 5**, Cross only captures useful cross-branch information, but JK only is a strong aggregation control. The clearest positive pattern appears when cross-residual exchange is paired with multi-layer aggregation and residual filtering or transformation. Cross + JK with learnable-threshold filtering gives the highest random AUPRC and AUROC among the strict controls (0.9312 ± 0.0010 and 0.9487 ± 0.0006). This supports a conditional architectural interpretation: cross-residual exchange is most useful when it is coupled with JK aggregation and residual filtering or transformation, where it enables additional information flow between the structural and relation-aware branches.

In the cold-gene half, Cross + JK with an MLP residual transform gives the highest AUPRC and AUROC (0.9050 ± 0.0031 and 0.9294 ± 0.0014). The margins over JK only are small, but the direction of the ranking metrics indicates that transformed cross-branch corrections can add useful signal when positive supervision for test genes is withheld.

### 3.4 Ablation and diagnostic analyses

#### 3.4.1 Robustness to negative sampling ratio

The cold-gene setting becomes more informative when the number of sampled unknown gene–trait pairs changes. **Figure 5** and **Table 4** summarize negative ratios from 0.25 to 5.0. AUPRC is prevalence-sensitive, so comparisons are interpreted within each ratio rather than across ratios.

At the low negative ratio of 0.25, Cross + JK with learnable-threshold filtering gives the highest coldgene AUPRC and AUROC among the compared methods, with AUPRC 0.9749 ± 0.0015 and AUROC 0.9321 ± 0.0025. Relative to MLP at the same ratio, the gains are +0.0494 AUPRC and +0.1357 AUROC. At ratios 0.5 and 1.0, MLP remains stronger by AUPRC, although Cross + JK variants remain close by AUROC. At harder ratios 3.0 and 5.0, MLP shows large performance fluctuations, whereas Cross + JK variants remain substantially above MLP and RGAT, and the MLP-residual transform gives the highest AUPRC and AUROC at the 5.0 ratio. These results preserve the default cold-gene benchmark ordering while adding a second perspective: Cross + JK provides robust information gain when candidate generation becomes more uncertain and the sampled negative distribution changes.

#### 3.4.2 Auxiliary context, feature-prior, and graph diagnostics

We next evaluate whether non-target biological relations can provide more than message-passing context by adding held-out typed-edge completion as a relation-balanced auxiliary objective. Auxiliary targets are removed from the message-passing graph, negatives are sampled within the correct source and destination types, and relation losses are averaged before weighting. **Table 6** shows the cold-gene effect. Relation balanced supervision improves JK only from 0.9019 to 0.9057 AUPRC and sparse cross-residual learning from 0.8994 to 0.9045 at the most favorable tested weights. The gains are small, but they indicate that auxiliary biological relations can help unseen-gene ranking when the losses are balanced by relation type.

**Table 6:** Cold-gene auxiliary supervision and biological context analyses.

Analysis	Model/setting	AUPRC	AUROC
No auxiliary loss	JK only	0.9019 ± 0.0030	0.9285 ± 0.0017
Relation-balanced auxiliary loss, *λ* = 0.50	JK only	0.9057 ± 0.0034	0.9293 ± 0.0020
No auxiliary loss	Sparse cross-residual	0.8994 ± 0.0059	0.9279 ± 0.0030
Relation-balanced auxiliary loss, *λ* = 0.05	Sparse cross-residual	0.9045 ± 0.0036	0.9297 ± 0.0017
Original graph	Cross + JK, learnable threshold	0.9023 ± 0.0029	0.9282 ± 0.0021
GO-similarity context	Cross + JK, learnable threshold	0.9107 ± 0.0052	0.9315 ± 0.0018
Original graph	Cross + JK, MLP residual	0.9050 ± 0.0031	0.9294 ± 0.0014
GO-similarity context	Cross + JK, MLP residual	0.9092 ± 0.0057	0.9316 ± 0.0023
Combined biological context	Cross + JK, MLP residual	0.9054 ± 0.0038	0.9290 ± 0.0030

Values are mean ± standard deviation over five seeds.

We also evaluated biologically derived context augmentation for cold-gene transfer. GO-similarity edges improve Cross + JK with learnable-threshold filtering from 0.9023 to 0.9107 AUPRC and improve the MLP residual Cross + JK variant from 0.9050 to 0.9092. Combining all added context edges is not uniformly better, suggesting that relation quality and redundancy matter more than simply increasing graph density.

Feature-prior and graph-randomization diagnostics provide complementary controls. Degree/isolationonly MLP reaches 0.9191 random AUPRC and 0.9133 cold-gene AUPRC, whereas membership-only and no-degree variants fall near random ranking, confirming that simple topological priors explain an important part of the feature-only signal. In contrast, dual-branch graph models remain strong with constant or reduced node features; for example, late fusion with constant features reaches 0.9155 random AUPRC and 0.9004 cold-gene AUPRC. This indicates that graph topology itself carries substantial signal, even when feature priors are removed.

The randomization controls also constrain the biological interpretation. Target-label shuffling sharply degrades performance, confirming that the pipeline is learning from supervision rather than producing rankings independently of labels. Feature shuffling primarily affects MLP, while typed degree-preserving rewiring and relation-label shuffling produce more modest average changes for graph models. Relation level ablation similarly shows relation-dependent effects rather than uniform gains from every biological edge family. The ranked pairs should therefore be interpreted as graph-supported candidate hypotheses, not as independent biological validation.

### 3.5 Summary of experimental evidence

The final experimental picture supports a focused model claim. Cross-residual exchange is most favorable when combined with JK aggregation and residual filtering, and the Cross + JK family shows its clearest information gain in random-split ranking, cold-gene ranking under shifted negative-sampling regimes, and GO-based context augmentation. Across the main benchmark, the proposed Cross + JK variants are consistently placed among the leading methods across AUPRC, AUROC, macro-F1, and accuracy. Their random-split standard deviations are also among the smallest in the table, suggesting that the cross-residual architectures learn reliably across repeated seeds rather than depending on a single favorable run. The broader benchmark also shows important boundaries: MLP remains strongest by AUPRC in the default cold-gene split, RotatE remains a competitive KG-embedding baseline in the random-split setting, and some graph-Transformer variants are competitive under harder negative ratios. The contribution is therefore best stated as Cross + JK providing robust information gain in more uncertain candidate-ranking regimes, with explicit evidence about when the mechanism is beneficial and where simpler priors remain strong.

## 4 Discussion

The experiments clarify the role of cross-residual exchange. Its most favorable configurations combine layer-wise exchange with JK-style multi-layer aggregation and residual filtering or transformation. These configurations provide the clearest information gain within the strict dual-branch controls, especially when candidate generation becomes less certain. The MLP-residual version further improves the cold-gene dualbranch mean, suggesting that cross-branch corrections are useful when they are selectively transformed rather than simply accumulated.

This distinction also clarifies the relationship between cross-residual exchange and fusion. Late fusion and JK-style aggregation combine branch or layer representations at readout, whereas cross-residual exchange modifies each branch during propagation. Their effects are complementary rather than interchangeable. The Cross + JK result indicates that intermediate interaction can coexist with a strong final aggregation mechanism.

The expanded benchmark supports a cautious interpretation. Cross + JK with learnable-threshold filtering gives the highest observed mean in the random-split table, whereas MLP remains strongest by AUPRC in the default cold-gene split. However, the negative-ratio analysis changes the practical perspective: when the sampled candidate distribution shifts, MLP becomes substantially less stable, while Cross + JK variants provide robust information gain in more uncertain candidate-ranking regimes. RotatE remains a competitive KG-embedding reference point, RGAT approaches the strongest random-split neural results, and the GraphGPS-style variant is competitive under cold-gene transfer and becomes strong when the sampled negative pool is larger. Feature ablations show that degree and isolation priors account for much of the MLP signal. Graph models retain strong performance with constant features, but typed rewiring and relation-label shuffling cause only limited average degradation. Thus, graph topology contributes useful information, yet the current evidence for fine-grained relation semantics is conditional rather than comprehensive.

The held-out relation-balanced multi-task experiment directly addresses whether auxiliary biological relations improve gene–trait prediction. Auxiliary supervision produces small mean increases for the tested dual-branch models in the cold-gene setting, including an increase of approximately 0.0051 AUPRC for sparse cross-residual learning at *λ*_aux_ = 0.05. The cross-residual setting is therefore not weak under relation-balanced supervision; rather, it can gain useful signal when auxiliary edge losses are balanced by relation type. GO-based context augmentation also improves the Cross + JK variants, whereas combining all added context edges is not uniformly better. These patterns are compatible with the view that auxiliary biological context can help unseen-gene ranking, but relation quality and loss balancing matter.

The study has several limitations. Trait normalization and grouping determine graph semantics and positive labels, so alternative phenotype ontologies may change both the task and the ranking results. Sampled unobserved pairs are not verified negative associations. The graph is rice-centered, auxiliary relation quality is uneven, and the five-seed paired tests have limited power for small architectural differences. Finally, biological case studies mostly trace evidence already connected to the integrated resources and should not be treated as independent experimental validation.

Future work should prioritize ontology-aligned trait normalization, relation-confidence calibration, external or cross-resource validation, and multi-task objectives with task-adaptive weighting. These directions are likely to strengthen the biological specificity of cross-residual exchange more effectively than adding architectural complexity alone.

## 5 Conclusion

We present a cross-residual knowledge-graph learning framework for robust multi-trait gene–trait prioritization in rice and evaluate it against feature-only, graph, heterogeneous attention, graph-Transformer, KG-embedding, multi-task, and dual-branch controls under random and cold-gene splits. Cross + JK with learnable-threshold filtering gives the highest observed mean in the random-split benchmark table, and the broader comparison identifies both graph-based information gains and strong feature-prior effects under cold-gene transfer.

Within the dual-branch family, Cross + JK variants achieve the highest mean AUPRC in both splits, and the MLP-residual form gives the strongest cold-gene dual-branch result. The clearest favorable evidence appears in negative-ratio sensitivity and GO-based context augmentation: MLP is strong under the default cold-gene sampled-candidate setting, but becomes unstable when the sampled candidate distribution shifts, whereas Cross + JK variants provide robust information gain in more uncertain candidate-ranking regimes. Overall, the results support filtered cross-branch interaction as a useful mechanism for integrating heterogeneous biological context, while also identifying the regimes in which strong feature priors or alternative graph models remain sufficient.

## Data Availability

The original contributions presented in the study are included in the article/supplementary material. Further inquiries can be directed to the corresponding author.
